# Numerical analysis of magnetohydrodynamics Casson nanofluid flow with activation energy, Hall current and thermal radiation

**DOI:** 10.1038/s41598-023-28379-5

**Published:** 2023-03-10

**Authors:** Y. Suresh Kumar, Shaik Hussain, K. Raghunath, Farhan Ali, Kamel Guedri, Sayed M. Eldin, M. Ijaz Khan

**Affiliations:** 1 Department of Mathematics, Koneru Lakshmaiah Education Foundation, R.V.S Nagar, Moinabad-Chilkur Rd, Near AP Police Academy, Aziznagar, Hyderabad, 500075 Telangana India; 2 Department of Mechanical Engineering, Malla Reddy Engineering College, Hyderabad, Telangana India; 3 Department of Humanities and Sciences, St. Johns College of Engineering and Technology, Yemmiganur, Kurnool, Andhra Pradesh 518360 India; 4 Department of Mathematical Sciences, Federal Urdu University of Arts, Sciences & Technology, Gulshan-e-Iqbal, Karachi, 75300 Pakistan; 5 Mechanical Engineering Department, College of Engineering and Islamic Architecture, Umm Al-Qura University, P.O. Box 5555, Makkah, 21955 Saudi Arabia; 6 Center of Research, Faculty of Engineering, Future University in Egypt, New Cairo, 11835 Egypt; 7 Department of Mechanical Engineering, Lebanese American University, Beirut, Lebanon; 8 Department of Mathematics and Statistics, Riphah International University I-14, Islamabad, 44000 Pakistan

**Keywords:** Energy storage, Fossil fuels, Fuel cells

## Abstract

In this study we analyzed the flow, heat and mass transfer behavior of Casson nanofluid past an exponentially stretching surface under the impact of activation energy, Hall current, thermal radiation, heat source/sink, Brownian motion and thermophoresis. Transverse magnetic field with the assumption of small Reynolds number is implemented vertically. The governing partial nonlinear differential equations of the flow, heat and mass transfer are transformed into ordinary differential equations by using similarity transformation and solved numerically by using Matlab bvp4c package. The impact of each of the Hall current parameter, thermal radiation parameter, heat source/sink parameter, Brownian motion parameter, Prandtl number, thermophoresis parameter and magnetic parameter on velocity, concentration and temperature, is discussed through graphs. The skin friction coefficient along the x-and z-directions, the local Nusselt number and the Sherwood number are calculated numerically to look into the inside behavior of the emerging parameters. It is witnessed that the flow velocity is a diminishing function of the thermal radiation parameter and the behavior has observed in the case of Hall parameter. Moreover, mounting values of Brownian motion parameter reduce the nanoparticle concentration profile.

## Introduction

Non-Newtonian fluid theory is being widely adopted in view of its applicable features. A non Newtonian fluid exerts nonlinear relationships between the shear stress and rate of shear strain. In nature, a Non-Newtonian fluid acts as elastic solid, i.e. the flow does not occur with small shear stress. Casson fluid is one such model in Newtonian fluids. It is first invented by Casson^1^ in 1959. It is based on the structure of liquid phase and interactive behavior of solid of a two-phase suspension. Some examples of Casson fluid are Jelly, honey, tomato sauce and concentrated fruit juices. Human blood can also be treated as a Casson fluid in the presence of several substances such as fibrinogen, globulin in aqueous base plasma, protein, and human red blood cells. Squeezing flows are generated by natural stresses or vertical velocities of the moving boundary layer. The practical examples of squeezing flow are compression, polymer processing, and injection molding. Casson fluid is regarded as the most popular non-Newtonian fluid occupying a significant role in various fields such as bio- engineering operations chemical as well as mechanical applications. In the context of fluid mechanics, the study of Casson fluid flow was investigated by several scientists, engineers, mathematicians and researchers depending upon different situations. Keeping in view the various parameters on the flow properties of Casson fluid, a very recently Seth and Bhattacharyya^2^ have discussed Modeling and Numerical Simulation of hydromagnetic natural convection Casson fluid flow with Nth-Order chemical reaction and newtonian heating in porous medium. Seth et al.^3^ have discovered Double diffusive Magnetohydrodynamics Casson fluid flow in a non-Darcy porous medium with Newtonian heating and thermo-diffusion effects. Pramanik^4^ solved the problem based on Casson fluid flow past an exponentially porous stretching surface in presence of thermal radiation. Very recently Umavathi et al.^5^ have studied Magnetohydrodynamic squeezing Casson nanofluid flow between parallel convectively heated disks. Arshad Khan et al.^6^ studied Entropy generation and thermal analysis for rotary motion of hydromagnetic Casson nanofluid past a rotating cylinder with Joule heating effect. Naveenkumar et al.^7^ have studied Impact of thermophoretic particle deposition on heat and mass transfer across the dynamics of Casson fluid flow over a moving thin needle. Alhadhrami et al.^8^ studied Numerical simulation of local thermal non-equilibrium effects on the flow and heat transfer of non-Newtonian Casson fluid in a porous media. Kanayo et al.^9^ reviewed Muhammad, Double diffusive convection and cross diffusion effects on Casson fluid over a Lorentz force driven Riga plate in a porous medium with heat sink: An analytical approach. Jain and Parmar^10^ and Ganga et al.^11^ have examined the slip flow of Casson fluid over a stretching sheet. Raghunath and Obulesu^12^ have studied Unsteady Magnetohydrodynamics oscillatory Casson fluid flow past an inclined vertical porous plate in the presence of chemical reaction with heat absorption and Soret effects. Raghunath et al.^13^ have stuied Investigation of Magnetohydrodynamics Casson fluid flow past a vertical porous plate under the influence of thermal diffusion and chemical reaction. Recently, Senapati et al.^14^ have numerically investigated the Casson nanofluid flow over a stretching sheet.

Hall current is most prominent on the absolute value and orientation of the current density and thereby on the magnetic force term. Under the effects of Hall currents the convective flow problem with magnetic field is significant in view of engineering uses in electric transformers, transmission lines, refrigeration coils, power generators, Magnetohydrodynamics accelerators, nanotechnological processing, nuclear energy systems exploiting fluid metals, blood flow control and heating elements. In case of magnetic field of high strength and less density of the gas, the investigation of magnetohydrodynamic flows with Hall current have the best utilizations in the study of Hall accelerators and flight magnetohydrodynamic. Peristaltic flows have vast applications under the effects of applied magnetic field in the magnetohydrodynamic feature of blood, process of dialysis, oxygenation and hypothermia. Exploration of non-Newtonian fluid flows has been the focus of many scientists due to its vast applications in industries and engineering. Important applications are exist in food engineering, petroleum production, power engineering, in polymer solutions and in melt in the plastic processing industries. Hall effect plays an important role when the Hall parameter is high. Hall parameter is the ratio of electron cyclotron frequency to atom-electron collision frequency. Steady Magnetohydrodynamics boundary layer flow with free convection over a porous inclined plate was explored by Alam et al.^15^ with variable suction and Soret effect in the existence of Hall current. Eldahab^16^ studied the free convective Magnetohydrodynamics flow along with the Hall effects through a stretching sheet. Thamizsudar^17^ discussed the impact of Hall current and rotation on the heat and mass transfer of Magnetohydrodynamics fluid flowing over an exponentially accelerated vertical plate. Ibrahim and Anbessa^18^ investigated the mixed convection flow of nanofluid with Hall and ion-slip effects using spectral relaxation method. One dimensional unsteady Magnetohydrodynamics micropolar fluid flow with the effect of Hall current was analyzed by Islam et al.^19^. The chemically reactive second grade via porous saturated space was investigated by Raghunath et al.^20^ using a perturbation technique. Raghunath et al.^21^ have investigated the effects of Soret, Rotation, Hall, and Ion Slip on the unsteady flow of a Jeffrey fluid through a porous medium. Raghunath and Mohanaramana^22^ have researched Hall, Soret, and rotational effects on unsteady Magnetohydrodynamics rotating flow of a second-grade fluid through a porous media in the presence of chemical reaction and aligned magnetic field.

Thermal radiation plays an important role in dissipating heat from the surface. It has applications in manufacturing industries such as chopper, space vehicles, reliable equipment design, satellites, atomic furnaces, missiles, space technology and procedures related to high temperature. Jamshed e al.^23^ have studied Radiative heat transfer of second grade nanofluid flow past a porous flat surface: a single-phase mathematical model. Arshadkhan et al.^24^ reviewed Chemically reactive nanofluid flow past a thin moving needle with viscous dissipation, magnetic effects and hall current. Arshad khan et al.^25^ has reviewed Radiative swirl motion of hydromagnetic Casson nanofluid flow over rotary cylinder using Joule dissipation impact. Islam et al.^26^ have studied Radiative mixed convection flow of maxwell nanofluid over a stretching cylinder with joule heating and heat source/sink effects. Khan et al.^27^ have discussed Bio-convective and chemically reactive hybrid nanofluid flow upon a thin stirring needle with viscous dissipation.

The study of heat generation/absorption parameter on the moving fluid is influential in sight of diverse physical problems. Uneven heat generation plays crucial part in heat dissipation problems. With the accelerated development of electronic technology, efficient cooling of electronic equipment has evolved to cool a variety of electronic equipment and is provided by separate transistors for mainframes and power supplies for telephone switches. The influence of the heat generation/absorption plays a crucial role in the heat efficiency of base fluids. Its pertinence is seen in the heat discharge of nuclear fuel residues, food storage, the production of plastic and rubber sheets, motion of fluids in fixed bed reactors and much more. Recently, Raghunath et al.^28^ studied the heat absorption effects on dissimilar flow geometries. Kumar and Singh^29^ investigated impact of heat source/sink on Magnetohydrodynamics steady laminar boundary layer natural convective flow through a concentric annulus region directed vertically. Heat transfer, radiation, and heat source/sink effects on viscoelastic fluid on a stretching surface were analysed by Bataller^30^. Heat source and chemical reaction impact on Magnetohydrodynamics flow past a moving vertical plate with convective surface conditions are analysed by Dharmendar and Shankar^31^. Arshad Khan et al.^32^ studied Bio-convective micropolar nanofluid flow over thin moving needle subject to Arrhenius activation energy, viscous dissipation and binary chemical reaction. Naveen Kumar et al.^33^ studied Comprehensive study of thermophoretic diffusion deposition velocity effect on heat and mass transfer of ferromagnetic fluid flow along a stretching cylinder. Punith et al.^34^ have explored Impact of Binary Chemical Reaction and Activation Energy on Heat and Mass Transfer of Marangoni Driven Boundary Layer Flow of a Non-Newtonian Nanofluid. Naveed Khan et al.^35^ have studied Heat and mass transfer aspects of a transient bio-convective Maxwell nanofluid subject to convective boundary conditions with curved surface. Naveenkumar et al.^36^ have reviewed Heat transfer analysis in three-dimensional unsteady magnetic fluid flow of water-based ternary hybrid nanofluid conveying three various shaped nanoparticles: A comparative study. Varunkumar et al.^37^ expressed Exploration of Arrhenius activation energy on hybrid nanofluid flow over a curved stretchable surface. Ravisha et al.^38^ have possessed Penetrative ferroconvection in a heterogeneous Brinkman porous medium. Naveen Kumar et al.^39^ exploring the impact of magnetic dipole on the radiative nanofluid flow over a stretching sheet by means of KKL model. Punith Gowda et al.^40^ have discussed A Three-Dimensional Non-Newtonian Magnetic Fluid Flow Induced Due to Stretching of the Flat Surface With Chemical Reaction. Sarada et al.^41^ have studied Impact of exponential form of internal heat generation on water-based ternary hybrid nanofluid flow by capitalizing non-Fourier heat flux model. Sarada et al.^42^ discussed Effect of Magnetohydrodynamics on Heat Transfer Behaviour of a Non-Newtonian Fluid Flow over a Stretching Sheet under Local Thermal Non-Equilibrium Condition. Prasannakumara and Punith Gowda^43^ explored Heat and mass transfer analysis of radiative fluid flow under the influence of uniform horizontal magnetic field and thermophoretic particle deposition. Refs.^44–46^ indicates some advance research work in the field of material sciences^47–51^, highlights both analytical and numerical methods to tackle the highly nonlinear differential equation and material problems over a different geometries.

Present study is the extension work of Ibrahim and Anbessa^18^. In this study we analyzed the flow, heat and mass transfer behavior of Casson nanofluid past an exponentially stretching surface in presence of Hall current, thermal radiation, Brownian motion and thermophoresis, and heat source/sink. The governing partial nonlinear differential equations of the flow, heat and mass transfer are transformed into ordinary differential equations by using similarity transformation and solved numerically. The effects of various non-dimensional governing parameters on velocity, temperature and concentration profiles are discussed and presented graphically. Under some special conditions the results of the present study have an excellent agreement with existing studies.

## Formulation of the problem

**Figure 1:**
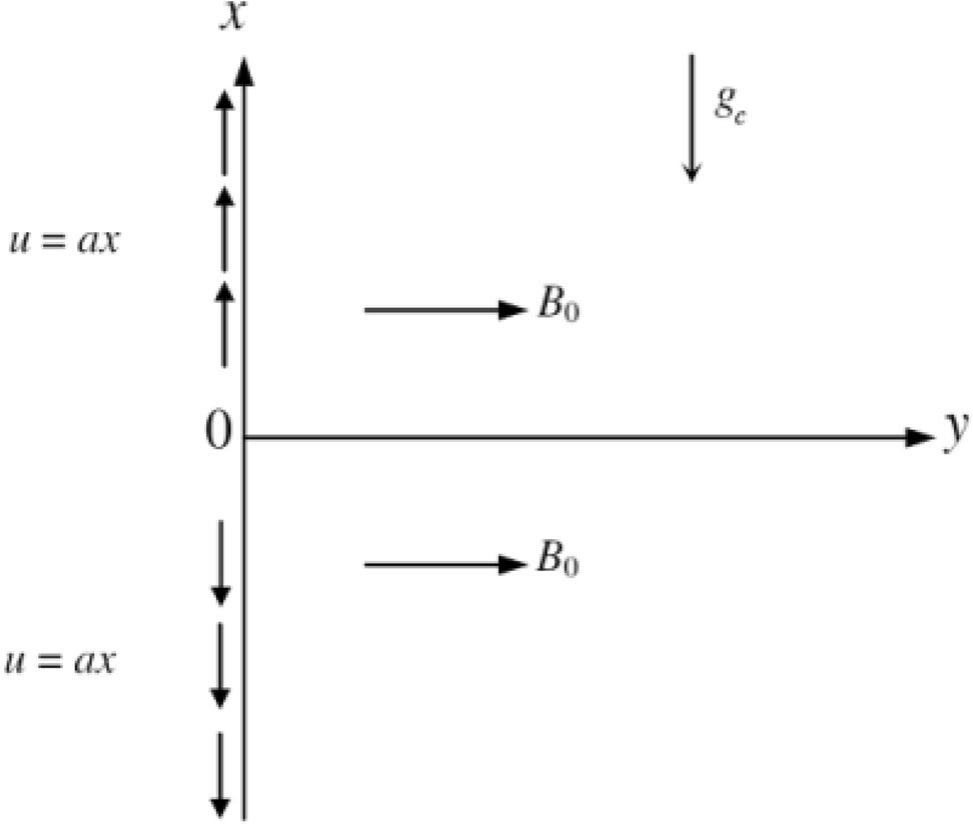
Physical configuration of the problem.

Here, steady heat and mass transfer of an incompressible hydromagnetic Casson nanofluid flow along a vertical stretching sheet coinciding with the plane y = 0, has been considered in the presence of the Hall current effects. By keeping the origin fixed, two opposite and equal forces are assumed to employ along the x-axis so that the sheet stretches linearly in both positive and negative direction (see Fig. 1). The governing equations and its corresponding boundary conditions followed by Ibrahim and Anbessa^18^.

i. With the assumption that the non-Newtonian nanofluid be electrically conducting and heat generating/absorbing, a strong magnetic field has been imposed normal to the direction of flow.
ii. Moreover, no electric field has been assumed to apply and the frequency of atom-electron collision has also been considered high for the generation of Hall current effect^52^.
iii. Due to the strong magnetic flux density B_0_, the Hall current effect is taken into consideration, however the small magnetic Reynolds number is employed and the induced magnetic field is ignored.
iv. Hall current effect is strong enough to give rise to a force in the z-direction and a cross flow is induced in the same direction which causes a flow.
v. It is further assumed that there are no variations in the flow, heat and mass transfer in the z-direction. This assumption can be achieved by taking the sheet of infinite width.
vi. Further, the effects of viscous dissipation and Joule heating are ignored.

By the above mentioned assumptions and Boussinesq approximation, the mathematical form of the problem is

1 $$\frac{\partial u}{{\partial x}} + \frac{\partial v}{{\partial y}} = 0$$

2 $$u\frac{\partial u}{{\partial x}} + v\frac{\partial u}{{\partial y}} = \nu \left( {1 + \frac{1}{\beta }} \right)\frac{{\partial^{2} u}}{{\partial y^{2} }} - \frac{{\sigma B_{0}^{2} }}{{\rho \left( {1 + m^{2} } \right)}}\left( {mw + u} \right) + g_{c} \beta_{T} (T - T_{\infty } )\, + g_{c} \beta_{C} (C - C_{\infty } )$$

3 $$u\frac{\partial w}{{\partial x}} + v\frac{\partial w}{{\partial y}} = \nu \left( {1 + \frac{1}{\beta }} \right)\frac{{\partial^{2} w}}{{\partial y^{2} }} + \frac{{\sigma B_{0}^{2} }}{{\rho \left( {1 + m^{2} } \right)}}\left( {mu - w} \right)$$

4 $$u\frac{\partial T}{{\partial x}} + v\frac{\partial T}{{\partial y}} = \frac{k}{{\rho C_{p} }}\frac{{\partial^{2} T}}{{\partial y^{2} }} + \frac{{Q_{0} }}{{\rho C_{p} }}(T - T_{\infty } )\, + \tau \left( {D_{B} \frac{\partial C}{{\partial y}}\frac{\partial T}{{\partial y}} + \frac{{D_{T} }}{{T_{\infty } }}\left( {\frac{\partial T}{{\partial y}}} \right)^{2} } \right) - \frac{1}{{\rho C_{p} }}\frac{{\partial q_{r} }}{\partial y}$$

5 $$u\frac{\partial C}{{\partial x}} + v\frac{\partial C}{{\partial y}} = D_{B} \frac{{\partial^{2} C}}{{\partial y^{2} }} + \frac{{D_{T} }}{{T_{\infty } }}\frac{{\partial^{2} T}}{{\partial y^{2} }} - k_{r}^{2} \left( {C - C_{\infty } } \right)\left( {\frac{T}{{T_{\infty } }}} \right)^{n} \exp \left( {\frac{{ - E_{a} }}{{\kappa T}}} \right)$$

The corresponding boundary conditions for the governing Partial differential equations are

6 $$\begin{aligned} & u = ax,\,\,\,\,\,v = 0,\,\,\,\,\,\,\,\,w = 0,\,\,\,\,\,\,\,\,\,\,\,T = T_{w} ,\,\,\,\,\,\,\,\,\,\,\,C = C_{w} \,\,\,\,\,\,\,\,\,\,\,\,\,\,\,\,\,\,\,\,\,\,\,\,\,\,\,\,{\text{at}}\,\,y = 0 \hfill \\ & u \to 0,\,\,\,\,\,\,\,\,\,w \to 0,\,\,\,\,\,\,\,\,\,T \to \,\,T_{\infty } \,\,\,\,\,\,\,\,\,\,C \to C_{\infty } \,\,\,\,\,\,\,\,\,\,\,\,\,\,\,\,\,\,\,\,\,\,\,\,\,\,{\text{as}}\,\,\,\,\,\,\,y \to \infty \hfill \\ \end{aligned}$$

The Rosseland approximation can be used for the radiative heat flux vector qr because there is also self-absorption in addition to emission for an optically thick fluid. Since the absorption coefficient is typically wavelength dependent and significant, we can use the Rosseland approximation. Therefore, the definition of qr is^35^.

7 $$q_{r} = \frac{{ - 4\sigma_{ 1 } }}{{3k^{ * } }}\frac{{\partial^{2} T^{4} }}{\partial y}$$

In this equation, k* denotes the Rosseland mean absorption co-efficient and σ_1_ stands for the Stefan–Boltzmann constant.

We are working under the assumption that the temperature changes inside the flow are not very significant, allowing us to describe T^4^ as a linear function. We extend T′^4^ about the free stream temperature T using Taylor's series, ignoring higher order variables in the process. The following is an approximation that may be derived from this:

8 $$T^{4} \approx 4T_{\infty }^{3} - 3T_{\infty }^{4}$$

The equation for energy (3) may be obtained by combining Eqs. (7) and (8), as shown in the following:

9 $$u\frac{\partial T}{{\partial x}} + v\frac{\partial T}{{\partial y}} = \frac{k}{{\rho C_{p} }}\frac{{\partial^{2} T}}{{\partial y^{2} }} + \frac{{Q_{0} }}{{\rho C_{p} }}(T - T_{\infty } )\, + \tau \left( {D_{B} \frac{\partial C}{{\partial y}}\frac{\partial T}{{\partial y}} + \frac{{D_{T} }}{{T_{\infty } }}\left( {\frac{\partial T}{{\partial y}}} \right)^{2} } \right) + \frac{{16\sigma_{ 1 } T_{\infty }^{3} }}{{3\rho C_{p} k^{ * } }}\frac{{\partial^{2} T}}{{\partial y^{2} }}$$

The similarity transformation used to transform the Partial differential equations to dimensionless ordinary differential equations

10 $$\begin{aligned} \eta & = \sqrt {\frac{a}{\nu }} y, u = ax\,f'(\eta ), v = -\sqrt {{a}{\nu }}f(\eta ),\;\;w = ax\,g(\eta ),\; \hfill \\ \varphi \left( \eta \right) & = \frac{{C - C_{\infty } }}{{C_{w} - C_{\infty } }},\;\theta \left( \eta \right) = \frac{{T - T_{\infty } }}{{T_{w} - T_{\infty } }} \hfill \\ \end{aligned}$$

Substitute Eq. (10) into Eqs. (2), (3), (5) and (9) yields to obtain the subsequent non dimensional equations

11 $$\left( {1 + \frac{1}{\beta }} \right)f^{\prime\prime\prime} + ff^{\prime\prime} - f^{{\prime}{2}} + Gr_{x}\theta \, + Gr_{c}\varphi \, - \frac{M}{{1 + m^{2} }}\left( {f^{\prime} + mg} \right) = 0$$

12 $$\left( {1 + \frac{1}{\beta }} \right)g^{\prime\prime} + fg^{\prime} - f^{\prime}g + \frac{M}{{1 + m^{2} }}\left( {mf^{\prime} - g} \right) = 0$$

13 $$\left( {1 + R\,} \right)\theta^{\prime\prime} + \Pr \,f\theta^{\prime} + \Pr \,N{b} \left( {\theta^{\prime}\,\varphi^{\prime} + \frac{{N{t} }}{{N{b} }}\theta^{{\prime}{2}} } \right) + \Pr Q\,\theta = 0$$

14 $$\varphi^{\prime\prime} + \Pr \,\,L{e\,} f\,\varphi^{\prime} + \frac{{N{t} }}{{N{b} }}\theta^{\prime\prime} - K_{E} \left( {1 + \theta } \right)^{n} \varphi \,\exp \left( {\frac{ - E}{{1 + \theta }}} \right) = 0$$

The correlated Dimensionless boundary conditions (BCs) are

15 $$\begin{aligned} & f\left( 0 \right) = 0,\,\,\,\,f^{\prime}\left( 0 \right) = 1,\,\,\,\,g\left( 0 \right) = 0,\,\,\,\,\,\,\theta \left( 0 \right) = 0,\,\,\,\,\,\,\,\varphi \left( 0 \right) = 1\,\,\,\,\,\,\,\,\,\,\,at\,\,\,\,\,\,\,\,\eta = 0 \hfill \\ & f^{\prime}\left( \eta \right) \to 0,\,\,\,\,\,\,g\left( \eta \right) \to 0,\,\,\,\,\,\,\theta \left( \eta \right) \to 0,\,\,\,\,\,\,\,\varphi \left( \eta \right) \to 0\,\,\,\,\,\,\,\,\,\,\,\,\,\,\,\,as\,\,\,\,\,\,\,\,\eta \to \infty \hfill \\ \end{aligned}$$

In the equations that do not include dimensions, the important parameters are defined as

16 $$\begin{aligned} M & = \frac{{\sigma B_{0}^{2} }}{\rho a},\,\,\Pr = \frac{\nu }{\alpha } = \frac{{\nu \rho C_{p} }}{k},\,\,\,L{e} = \frac{\alpha }{{D_{B} }},\,\,Q = \frac{{Q_{0} }}{{a\rho C_{p} }},\,\,Gr_{x} = \frac{{g_{c} \beta_{T} \left( {T_{w} - T_{\infty } } \right)}}{{a^{2} x}}, \hfill \\ N{b} & = \frac{{\tau D_{B} \left( {C_{w} - C_{\infty } } \right)}}{\nu },\,\,\,\,\,N{t} = \frac{{\tau D_{T} \left( {T_{w} - T_{\infty } } \right)}}{\nu T_{\infty } },\,\,\,Gr_{c} = \frac{{g_{c} \beta_{C} \left( {C_{w} - C_{\infty } } \right)}}{{a^{2} x}},\, \hfill \\ R & = \frac{{16\sigma_{ 1 } T_{\infty }^{3} }}{{3k k^{ * } }},\,K_{E} = \frac{{k_{r}^{2} }}{a},\,Re^{2} = \frac{{ax^{2} }}{\nu},\ E = \frac{{E{}_{a}}}{{\kappa T_{\infty } }} \hfill \\ \end{aligned}$$

## Physical quantities of interests

The local skin friction coefficient in the direction of x Cf_x_, and in the direction of z Cf_z,_ the local Nusselt number Nu_x_, and the local Sherwood number Sh_x_ are the physical quantities of relevance that influence the flow. These numbers have the following definitions:

17 $$C\,f_{x} = \frac{{2\tau_{wx} }}{{\rho \,\left( {ax} \right)^{2} }},\,\,\,\,Cf_{z} = \frac{{2\tau_{wz} }}{{\rho \,\left( {ax} \right)^{2} }},\,\,\,\,Nu_{x} = \frac{{xq_{w} }}{{k\,\left( {T_{w} - T_{\infty } } \right)}},\,\,\,Sh_{x} = \frac{{x\,j_{w} }}{{D_{B} \left( {C_{w} - C_{\infty } } \right)}}$$

where τwx, τwy, qw and jw are the wall skin friction, wall heat flux and wall mass flux respectively given by

18 $$\tau_{wx} = \mu \left[ {\frac{\partial u}{{\partial y}}} \right]_{y = 0} ,\,\,\,\,\,\,\tau_{wz} = \mu \left[ {\frac{\partial w}{{\partial y}}} \right]_{y = 0} ,\,\,\,\,q_{w} = - k\left[ {\frac{\partial T}{{\partial y}}} \right]_{y = 0}+q_r ,\,\,\,\,\,\,\,\,j_{w} = - D_{B} \left[ {\frac{\partial C}{{\partial y}}} \right]_{y = 0}$$

The coefficient of skin friction, the Nusselt number, and the Sherwood number are all expressed in their non-dimensional versions in terms of the similarity variable as follows:

19 $${\text{Re}}_{x}^{1/2} Cf_{x} = 2f^{\prime\prime}(0),\,\,\,\,\,\,\,{\text{Re}}_{x}^{1/2} Cf_{z} = 2g^{\prime}(0),\,\,\,\,{\text{Re}}_{x}^{1/2} Nu_{x} = - (1+R)\theta^{\prime}(0),\,\,\,\,{\text{Re}}_{x}^{1/2} Sh_{x} = - \varphi^{\prime}(0)\,$$

## Solution methodology

The non-linear ODE system (11–14), susceptible to constraints 15, was solved using the shooting technique for various values of the related parameters. We were able to figure out from the graphs that the behavior of the solutions does not change much when the value is greater than 8. Because of this, and based on the results of the computational experiments described above, we are considering using the range [0, 8] as the domain of the issue rather than the range [0,∞].We denote f by y_1_, g by y_4_, θ by y_6_ and φ by y_8_ for converting the boundary value problem (11–15) to the following initial value problem consisting of 9 first order differential equations.

20 $$y^{\prime}_{1} = y_{2} ,\,$$

21 $$y^{\prime}_{2} = y_{3} ,\,$$

22 $$y^{\prime}_{3} = \left( {1 + \frac{1}{\beta }} \right)\left( { - y_{1} y_{3} + y_{2}^{2} - Gr_{x} y_{6} + Gr_{c} y_{8} + \frac{M}{{1 + m^{2} }}\left( {y_{2} + my_{4} } \right) } \right),$$

23 $$y^{\prime}_{4} = y_{5} ,\,$$

24 $$y^{\prime}_{5} = \left( {1 + \frac{1}{\beta }} \right)\left( {y_{2} y_{4} - y_{1} y_{5} - Gr_{x} y_{6} + Gr_{c} y_{8} - \frac{M}{{1 + m^{2} }}\left( { - y_{4} + my_{2} } \right)} \right),$$

25 $$y^{\prime}_{6} = y_{7} ,\,$$

26 $$y^{\prime}_{7} = - \Pr \,y_{1} y_{7} - \Pr \,N{b} \left( {y_{9} y_{7} + \frac{{N{t} }}{{N{b} }}y_{7}^{2} } \right) - A^{ * } e^{ - \eta } - B^{ * } y_{6} ,\,$$

27 $$y^{\prime}_{8} = y_{9} ,\,$$

28 $$y^{\prime}_{6} = - \Pr \,\,L{e\,} y_{1} \,y_{9} - \frac{{N{t} }}{{N{b} }}y^{\prime}_{7} + y_{8} K_{E} \left( {1 + y_{6} } \right)^{n} \exp \left( {\frac{ - E}{{1 + y_{6} }}} \right),\,$$

## Results and discussion

To envision the effect of various physical parameters on tangential velocity f^i^(η), transverse velocity g(η), nanoparticle concentration φ(η) and temperature θ(η) profiles, Figs. 2, 3, 4, 5, 6, 7, 8, 9, 10, 11, 12, 13, 14, 15, 16, 17, 18, 19, 20, 21, 22, 23, 24, 25, 26, 27, are plotted. In all these computations, unless mentioned, otherwise we have considered Nb = 0.3, Nt = 0.7, P r = 0.71, Le = 0.6, β = 0.5, M = 0.5, m = 0.2, Grx = 0.5, Grc = 0.5, Q = 0.5, R = 1, E = 0.5

**Figure 2:**
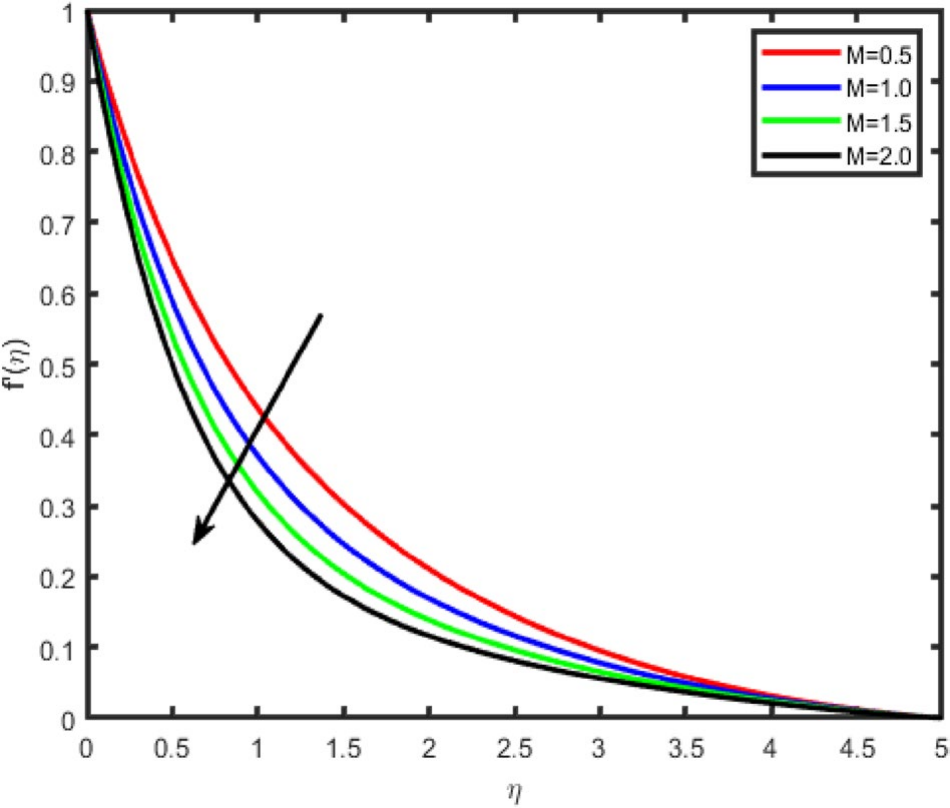
Effect of magnetic field parameter (M) on tangential velocity f′(η).

**Figure 3:**
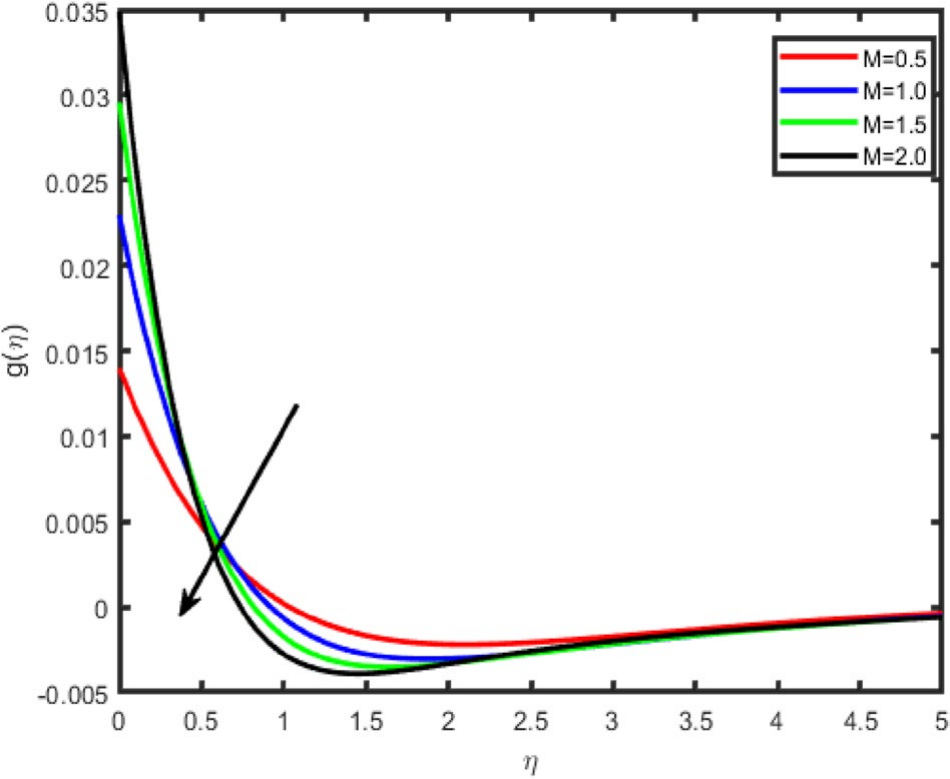
Effect of magnetic field parameter (M) on transverse velocity g(η).

**Figure 4:**
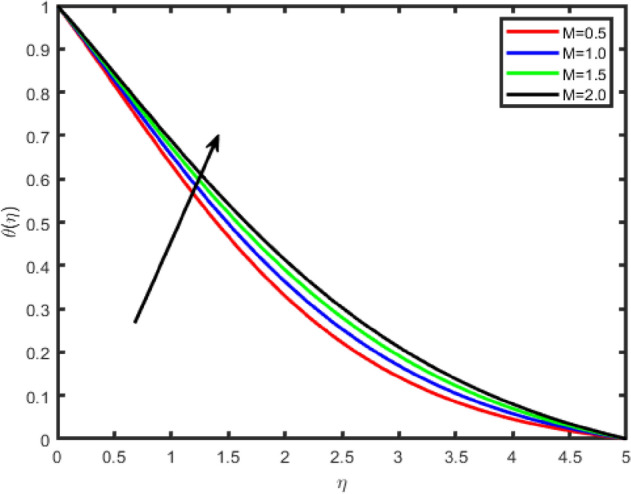
Effect of magnetic field parameter (M) on temperature θ(η).

**Figure 5:**
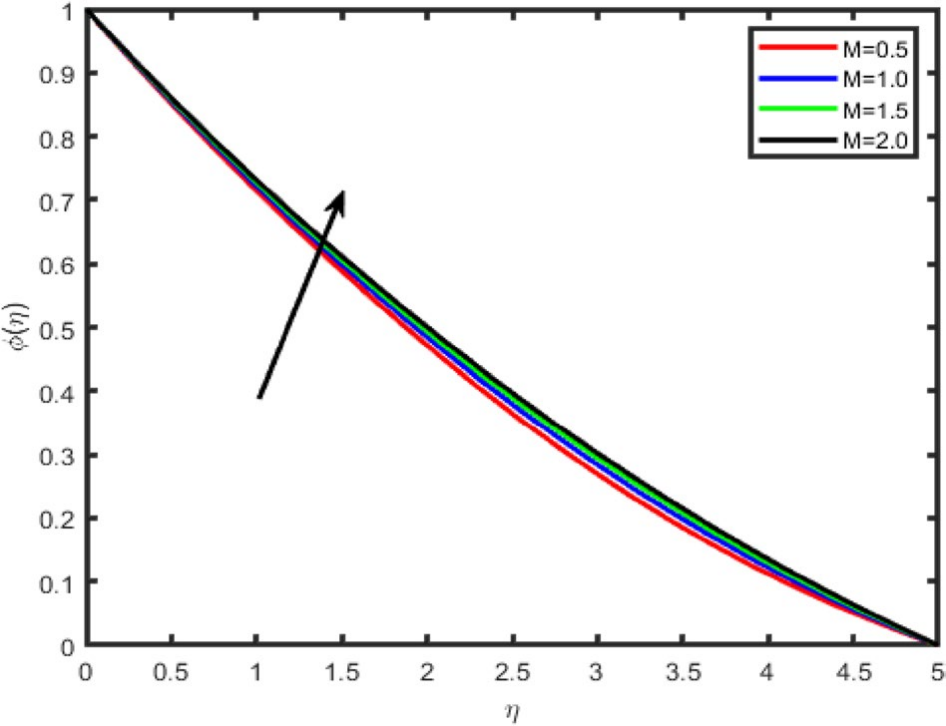
Effect of magnetic field parameter (M) on concentration ϕ(η).

**Figure 6:**
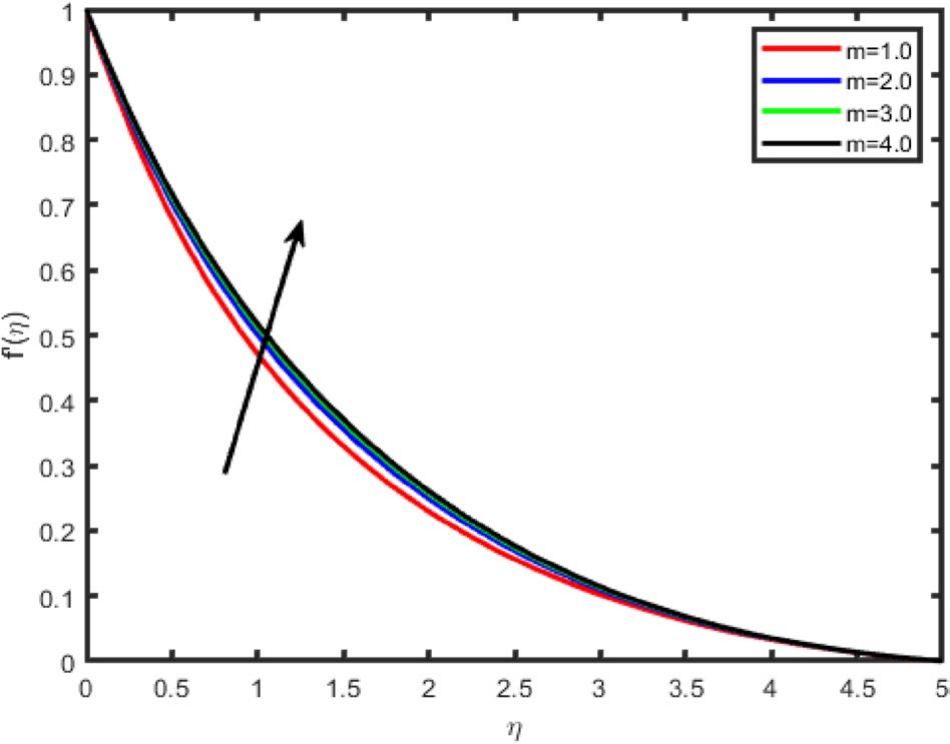
Effect of Hall parameter (m) on tangential velocity f′(η).

**Figure 7:**
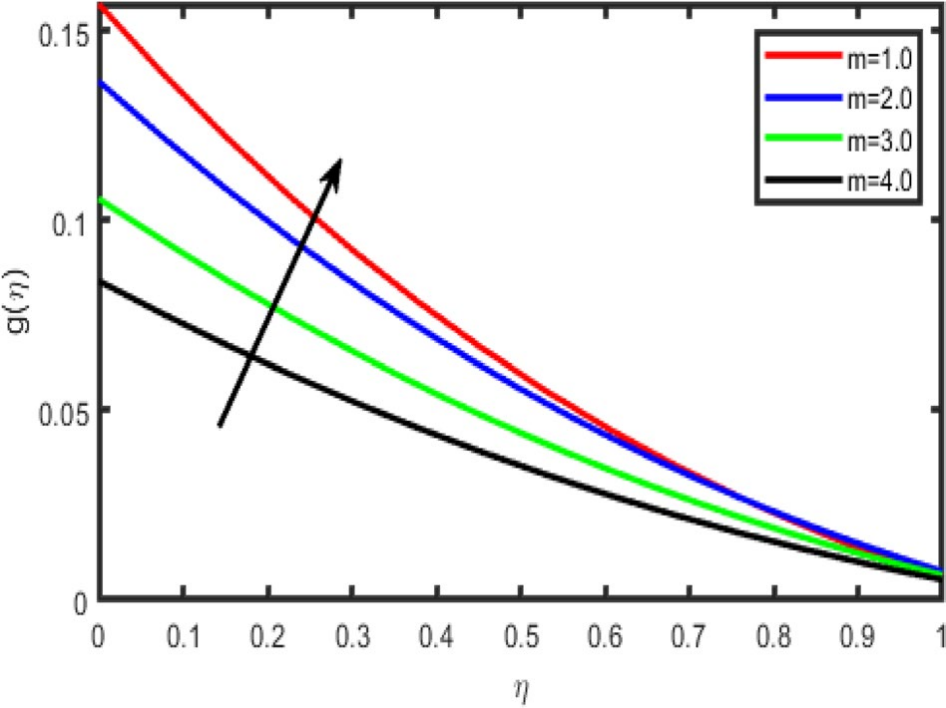
Effect of Hall parameter (m) on transverse velocity g(η).

**Figure 8:**
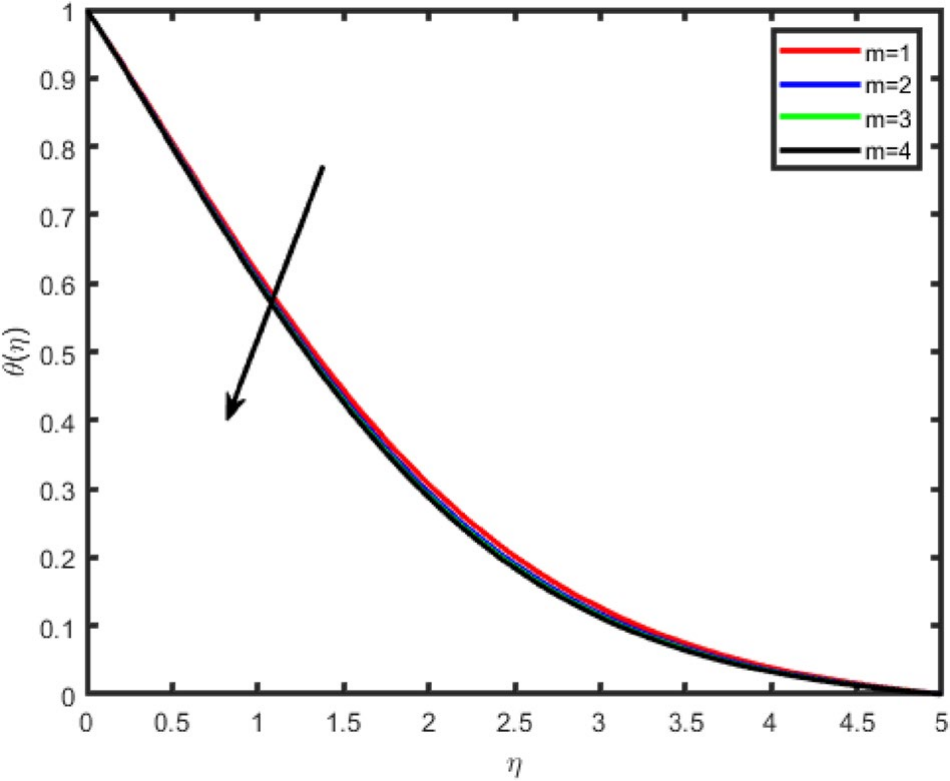
Effect of Hall parameter (m) on temperature θ(η).

**Figure 9:**
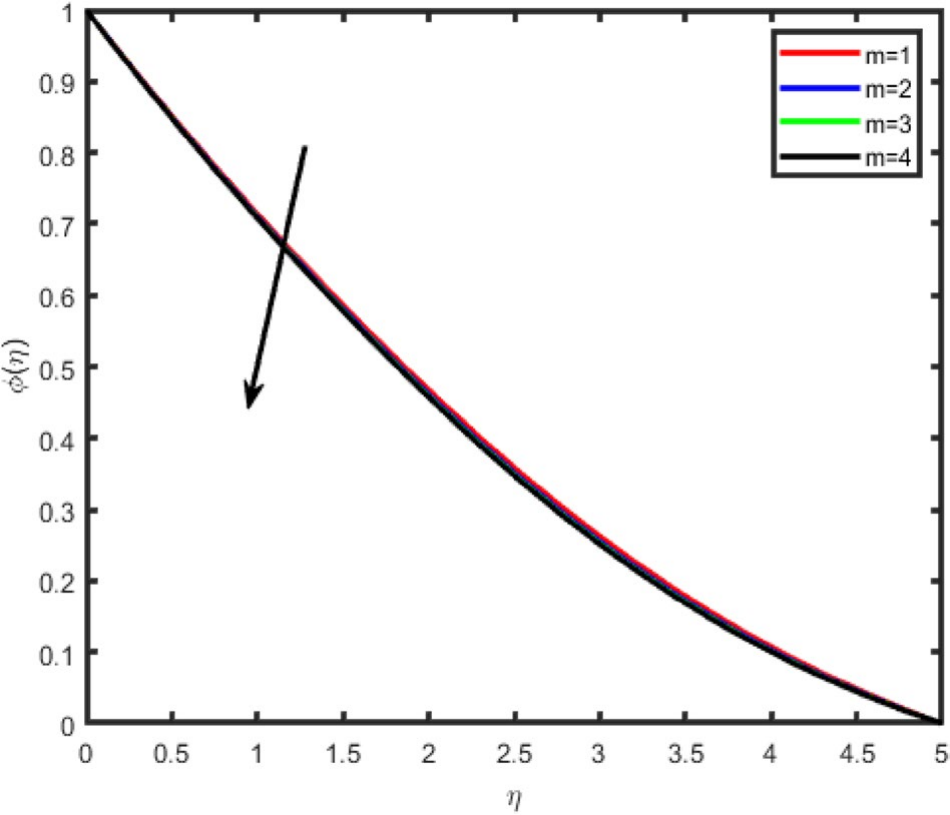
Effect of Hall parameter (m) on concentration ϕ(η).

**Figure 10:**
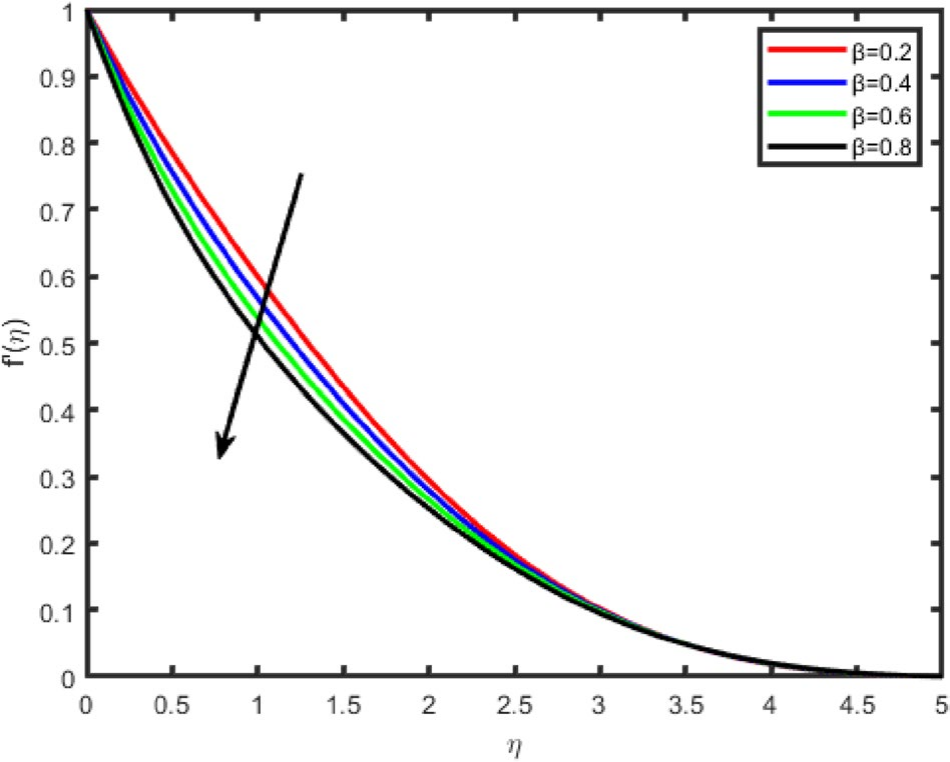
Effect of Casson fluid parameter (β) on tangential velocity f′(η).

**Figure 11:**
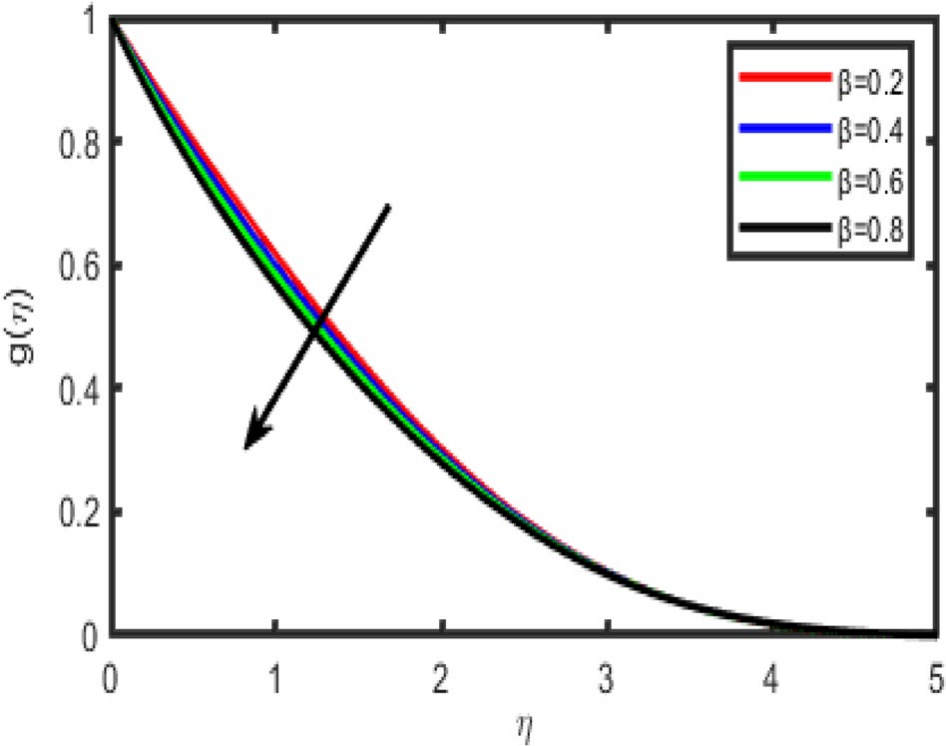
Effect of Casson fluid parameter (β) on transverse velocity g(η).

**Figure 12:**
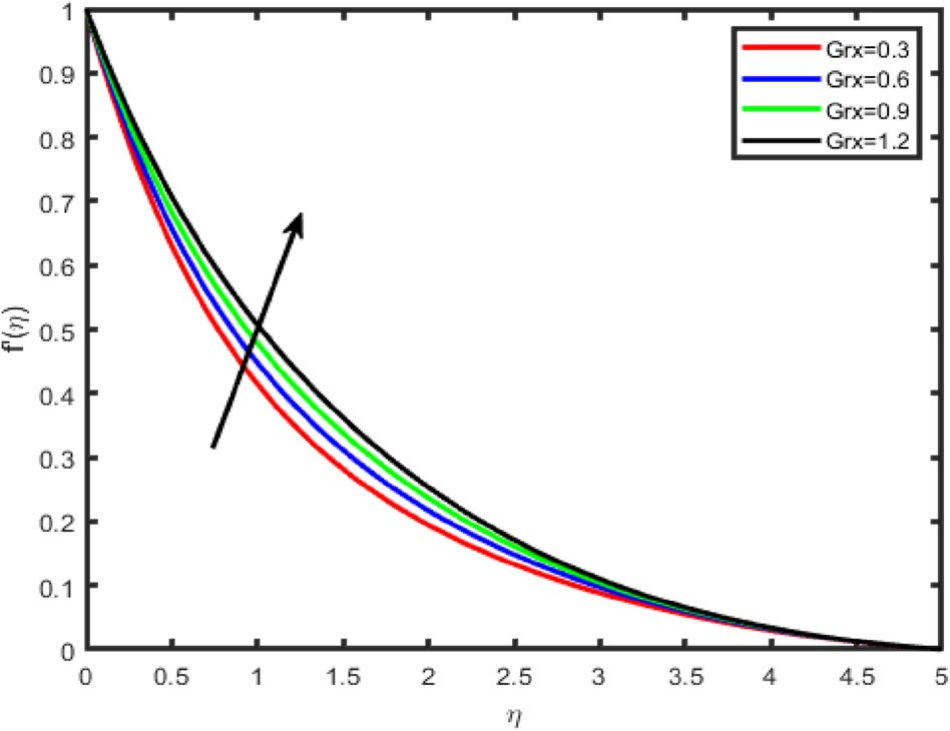
Effect of thermal Grashof number (Grx) on tangential velocity f′(η).

**Figure 13:**
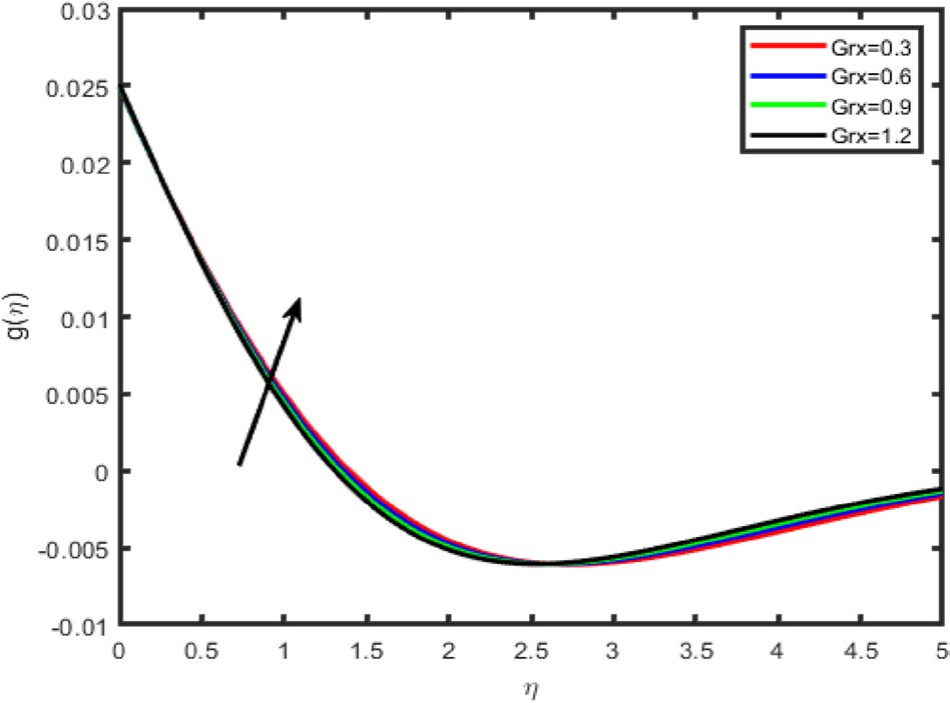
Effect of thermal Grashof number (Grx) on transverse velocity g(η).

**Figure 14:**
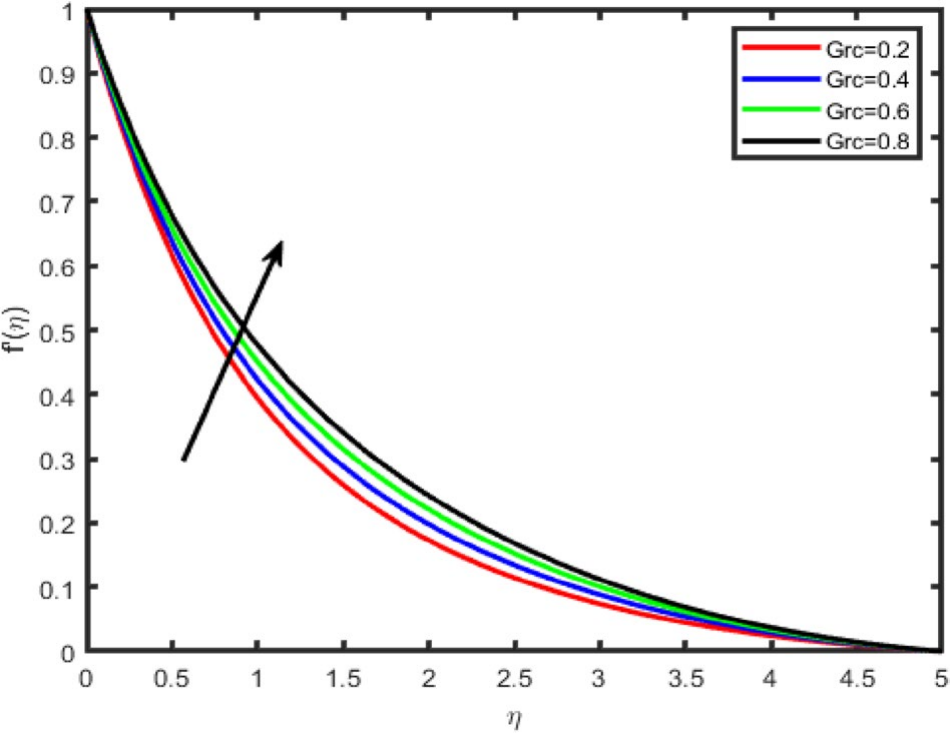
Effect of mass Grashof number (Grc) on tangential velocity f′(η).

**Figure 15:**
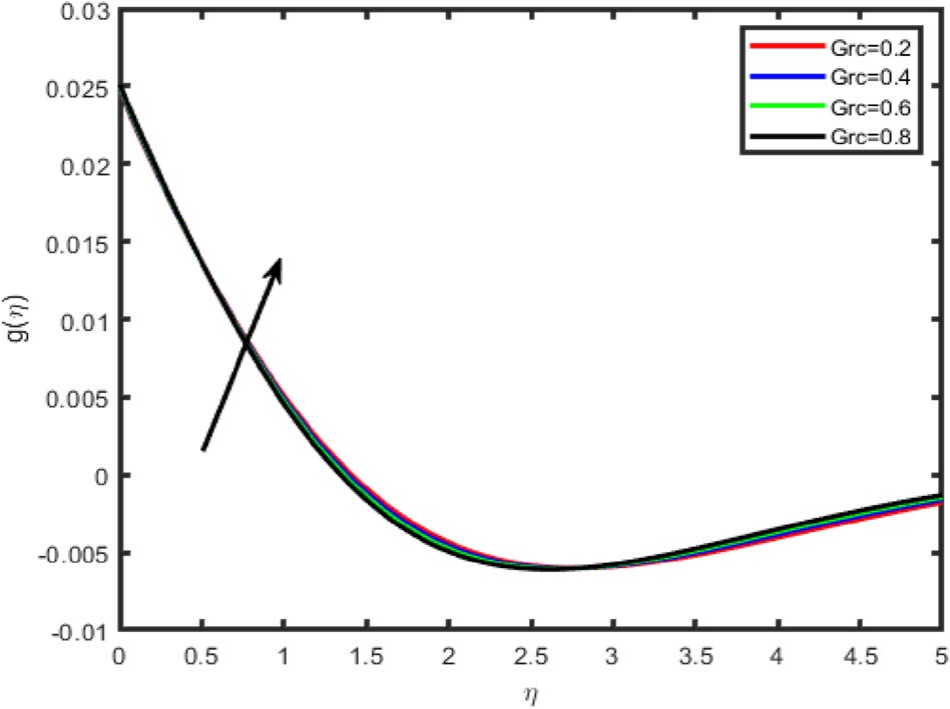
Effect of mass Grashof number (Grc) on transverse velocity g(η).

**Figure 16:**
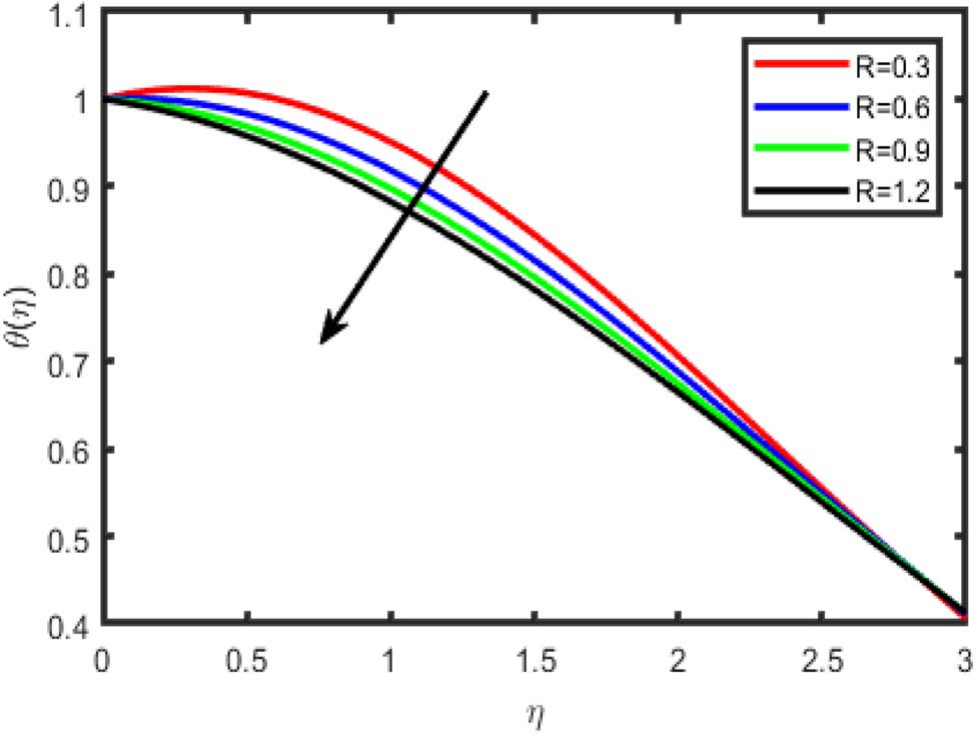
Effect of radiation parameter (R) on temperature θ(η).

**Figure 17:**
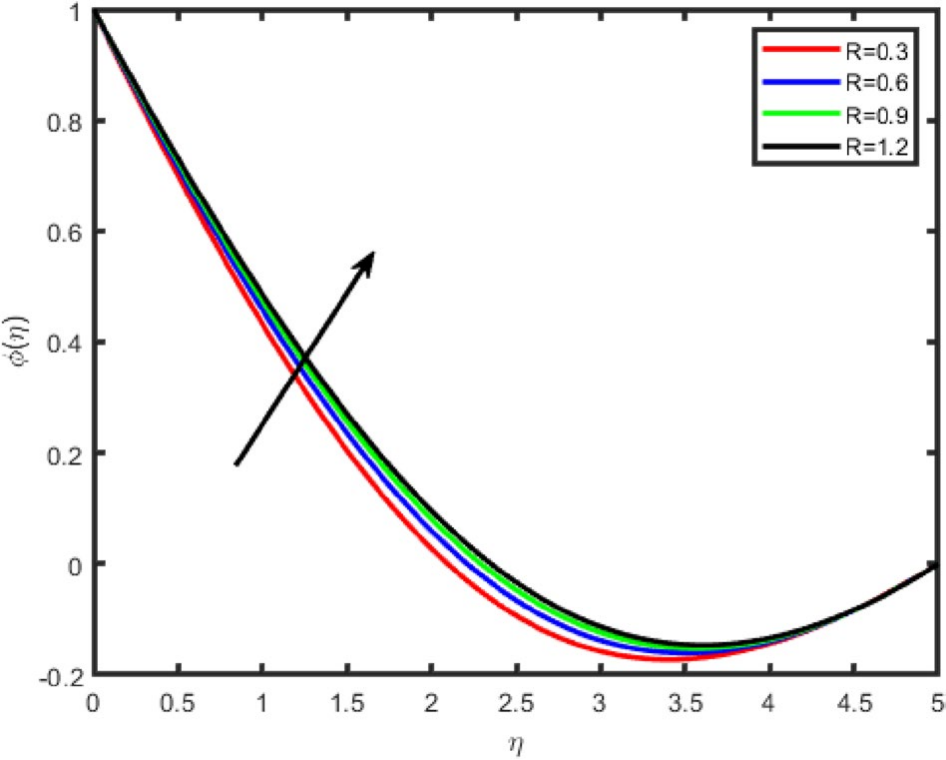
Effect of radiation parameter (R) on concentration ϕ(η).

**Figure 18:**
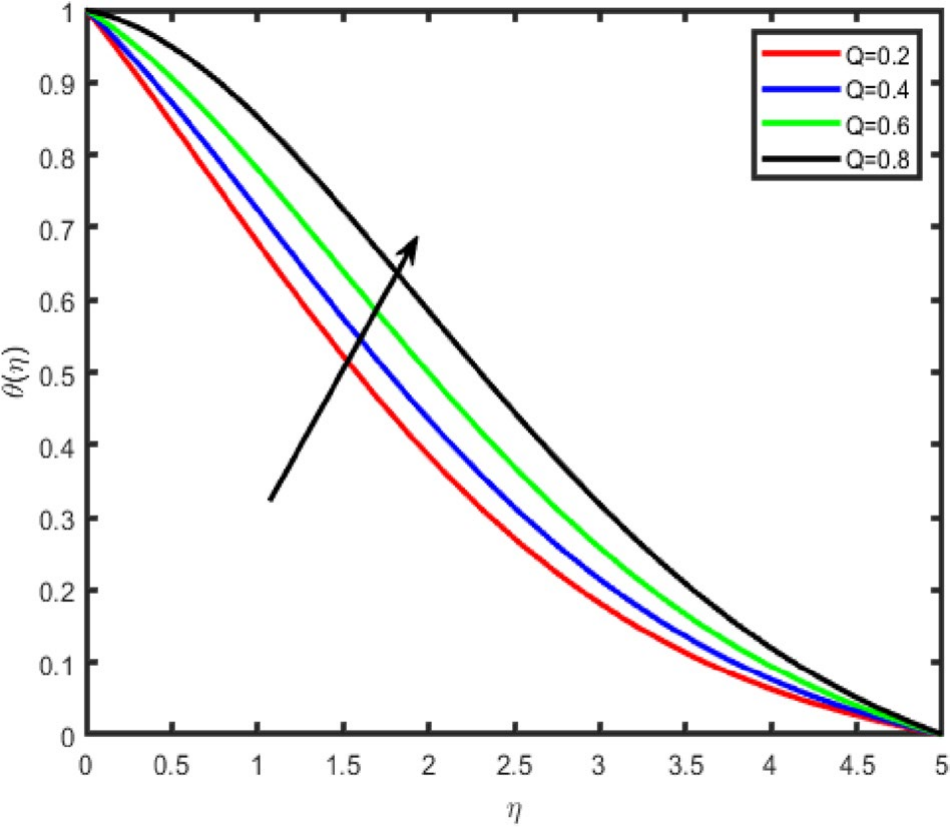
Effect of heat source parameter (Q) on temperature θ(η).

**Figure 19:**
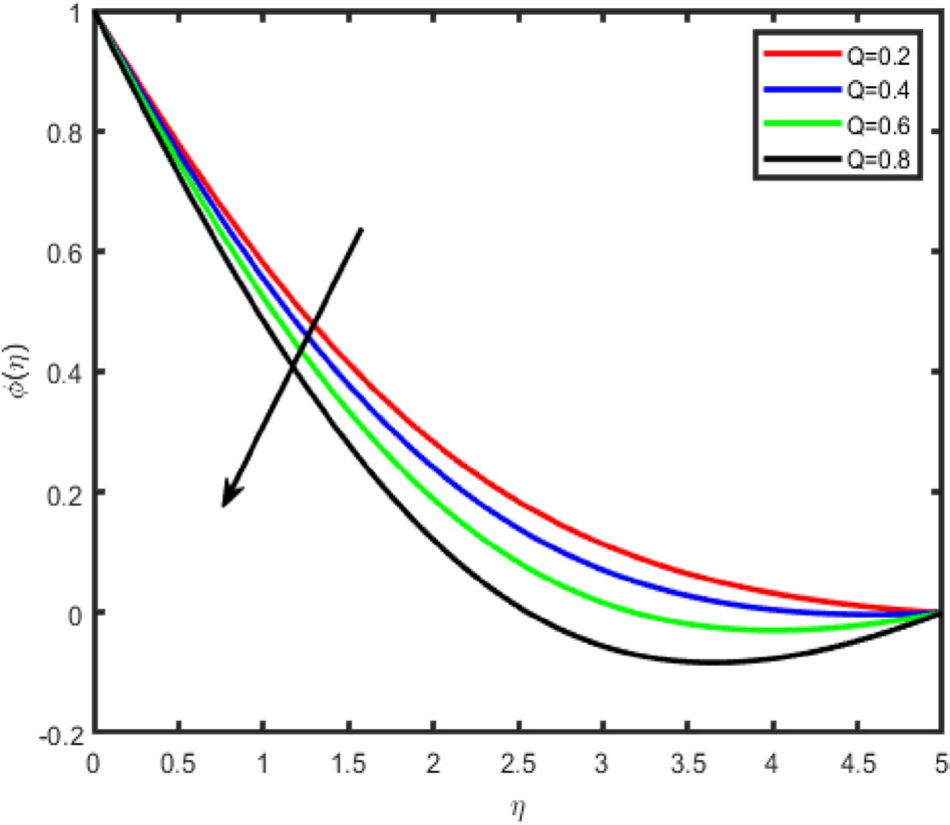
Effect of heat source parameter (Q) on concentration ϕ(η).

**Figure 20:**
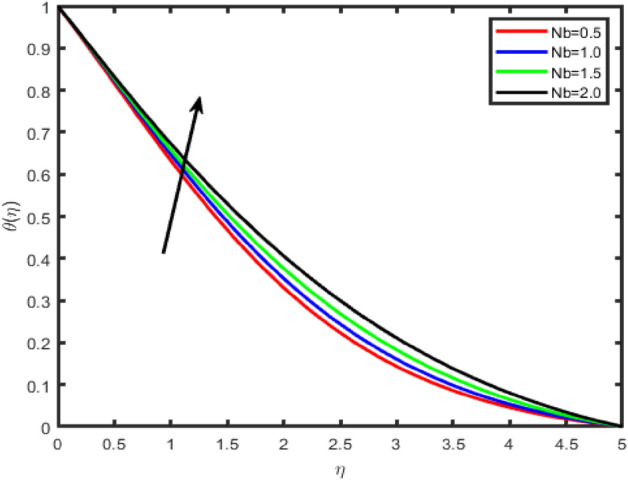
Effect of Brownian motion parameter (Nb) on temperature θ(η).

**Figure 21:**
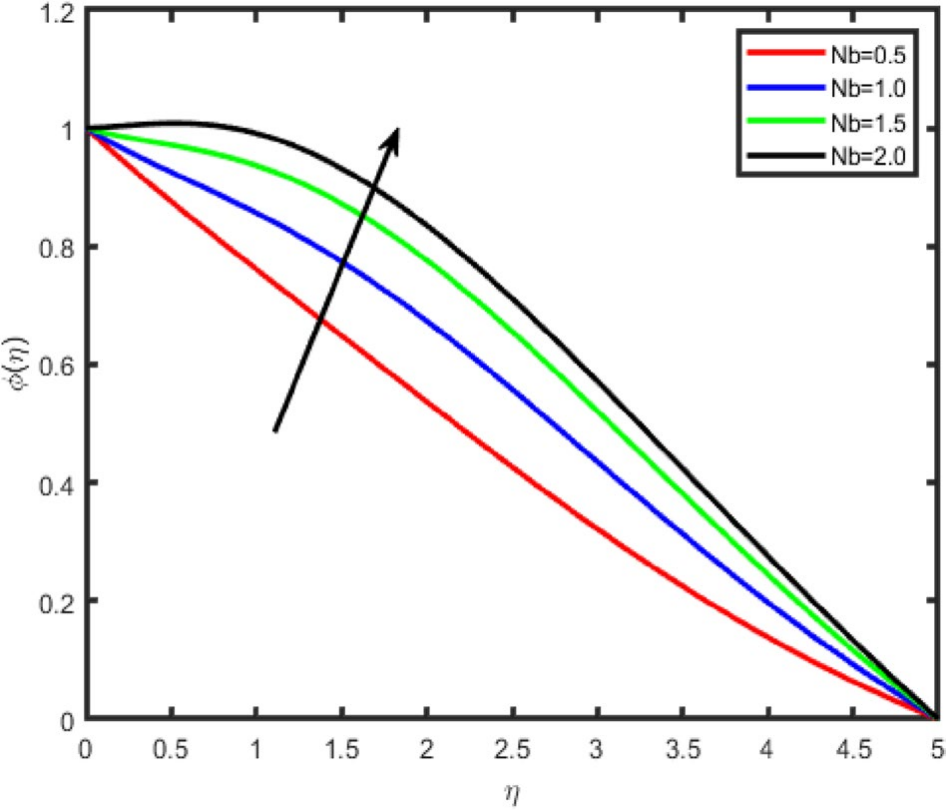
Effect of Brownian motion parameter (Nb) on concentration ϕ(η).

**Figure 22:**
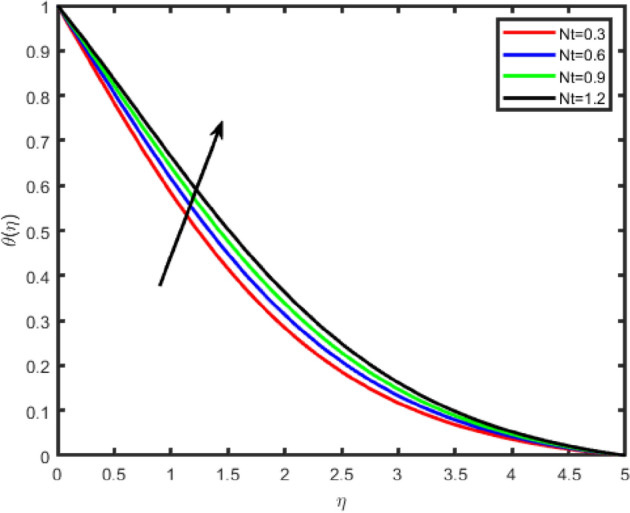
Effect of thermophoresis parameter (Nt) on temperature θ(η).

**Figure 23:**
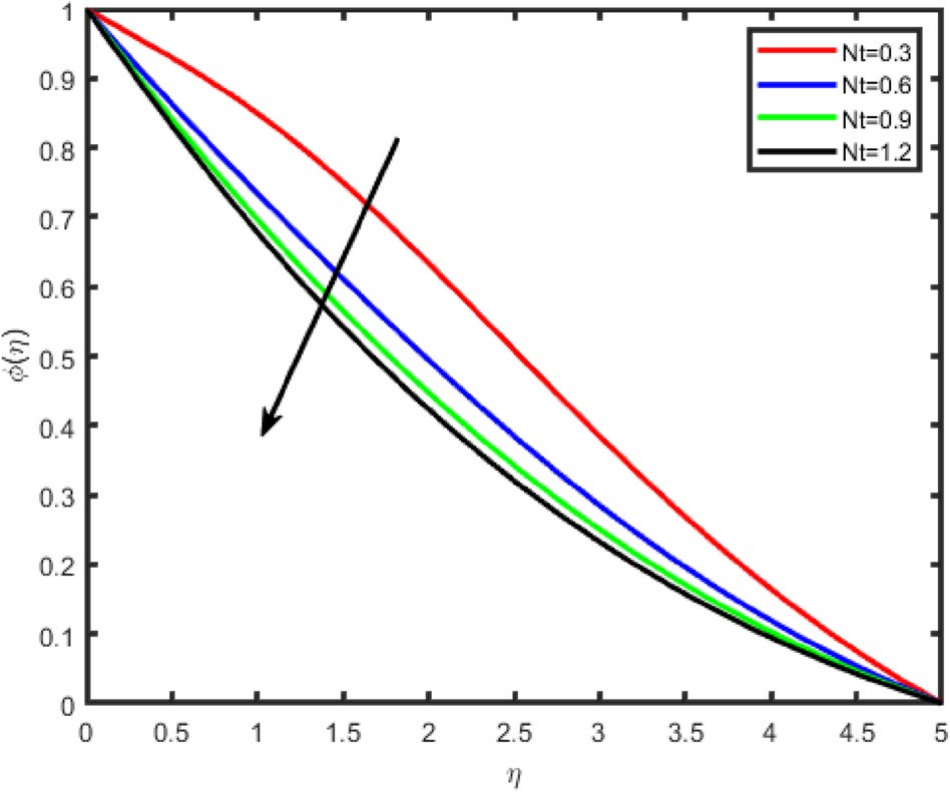
Effect of thermophoresis parameter (Nt) on concentration ϕ(η).

**Figure 24:**
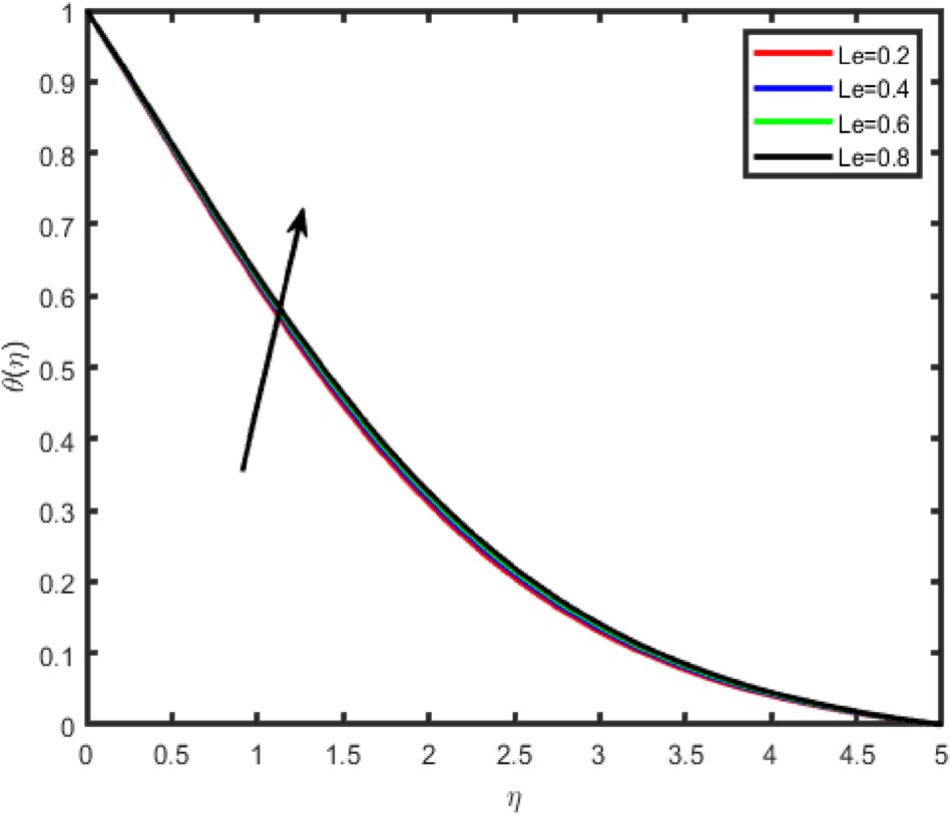
Effect of Lewis number (Le) on temperature θ(η).

**Figure 25:**
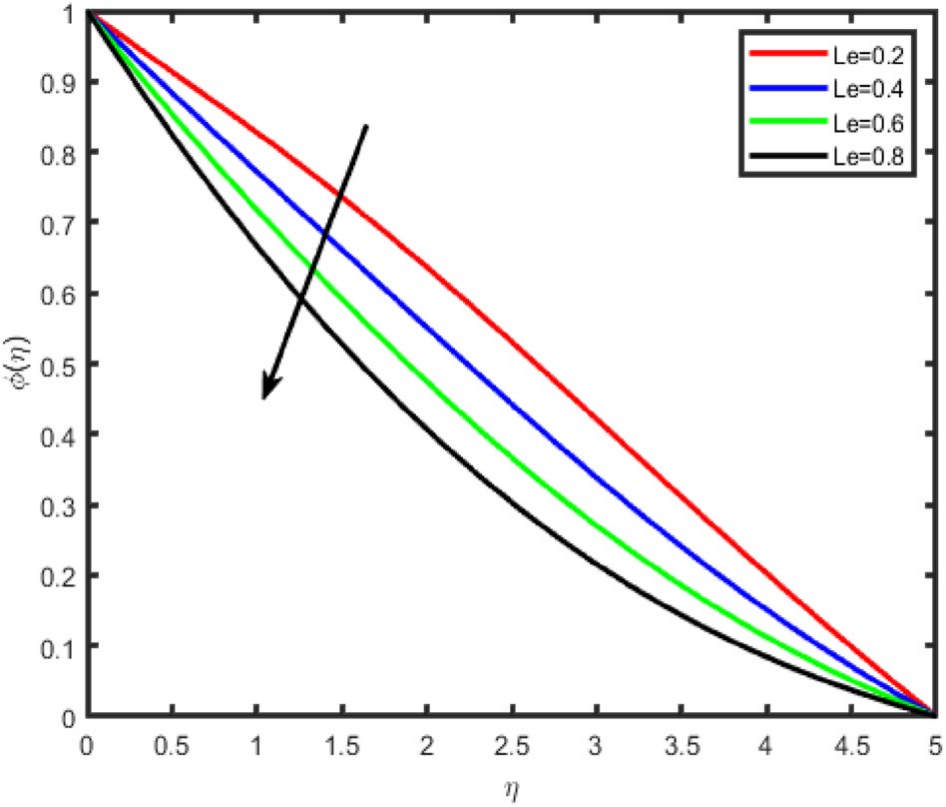
Effect of Lewis number (Le) on concentration ϕ(η).

**Figure 26:**
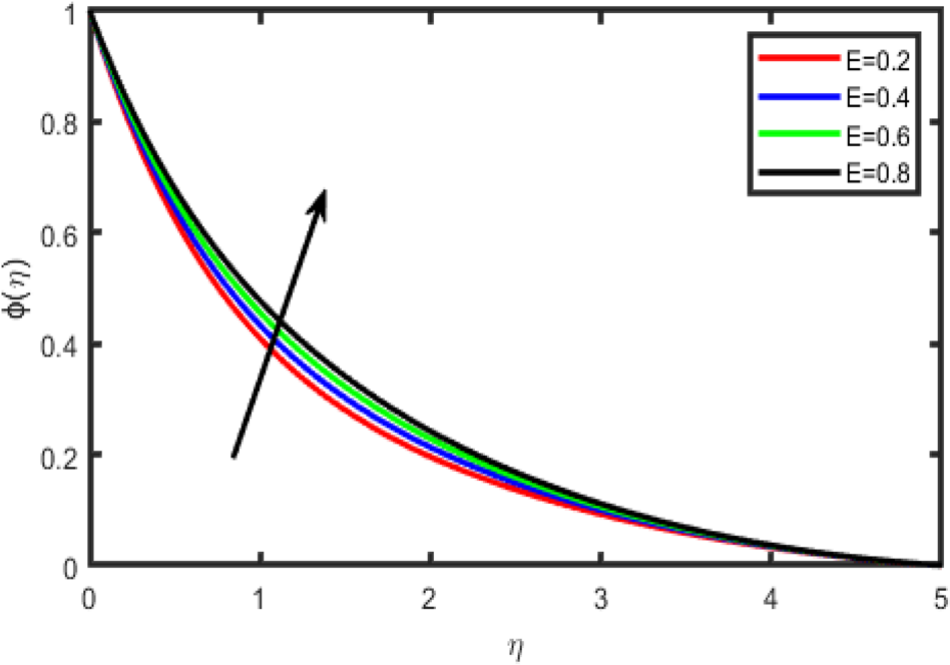
Effect of activation energy (E) on concentration ϕ(η).

**Figure 27:**
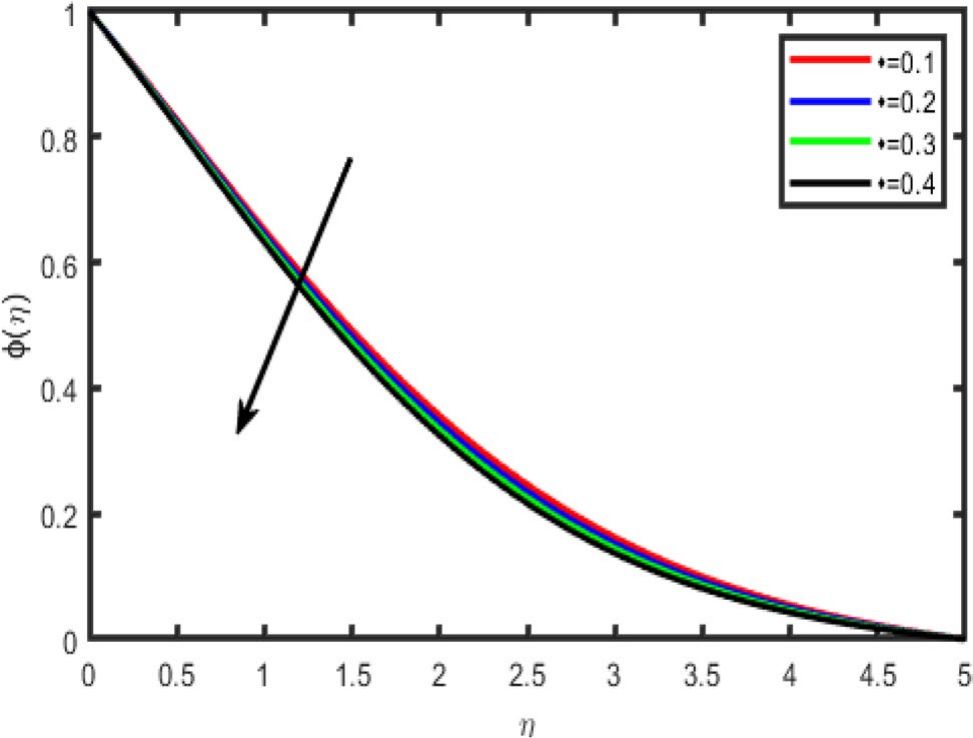
Effect of chemical reaction rate on concentration ϕ(η).

Figures 2, 3, 4, 5 shows the effect of magnetic parameter M on the tangential velocity f′(η), transverse velocity g(η), temperature θ(η), and concentration φ(η) profiles, respectively. The velocity profile f’(η) decreases with an increase in the values of M, the same behavior has observed transverse velocity g(η), and temperature θ(η) and concentration φ(η) profiles increase as M increases. As M increases, a drag force, called Lorentz force increases. Since this force opposes the flow of nanofluid, velocity in the flow direction decreases. Moreover, since an electrically conducting nanofluid with the strong magnetic field in the direction orthogonal to the flow are considered, an increase in M increases the force in the z-direction which results in an diminishes in the transverse velocity profile g(η).

Figures 6, 7, 8, 9 illustrate the impacts of the Hall parameter m on tangential velocity f^i^(η), transverse velocity g(η), nanoparticle concentration φ(η) and temperature θ(η) profiles, respectively. It is observed Figs. 6 and 7, the velocity f’(η) and g(η) profiles increase as m increases. But, the temperature and concentration profiles decrease with an increase in m as shown in Figs. 8 and 9. This is because the enclosure of Hall parameter decreases the resistive force caused by the magnetic field due to its effect of reducing the effective conductivity. Hence, the velocity component increases as the Hall parameter increases.

Figures 10 and 11 shows the effect of the Casson parameter (β) on the velocity profile. We notice that as β increases, the velocity and the boundary layer thickness decrease. Hence, the magnitude of the velocity is greater in Casson fluid when compared with viscous fluids.

In Figs. 12, 13, 14, 15 the effects of the thermal Grashof Gr and mass Grashof Gm numbers on the tangential velocity f′(η), the transverse velocity g(η), are displayed respectively. As the Grashof number is a ratio of the buoyancy force to the viscous force and it appears due to the natural convection flow, so an increase in the tangential velocity as well as the transverse velocity of the fluid. It happens because of the fact that higher the Grashof number implies higher the buoyancy force which means higher the movement of the flow. Figures 8 and 9 depict the influence of the solutal Grashof number on the temperature and the concentration profile respectively. An increase in the solutal Grashof number means a decrease in the viscous force which reduces the temperature and the concentration of the fluid.

Figures 16, 17 describes the behavior of thermal radiation parameter (R) on both temperature and concentration fields. It interesting to observe that for higher value of R strengthen the temperature because radiation parameter produces thermal energy in the flow region, therefore enhancement have been seen in the temperature field whereas reverse behaviour is seen for concentration.

Figure 18 shows that the temperature θ(η) increases with an increase in the resistance of the heat source/sink, due to an increase in the resistance of the heat generation, the temperature rises. The opposite behavior is observed in the case of concentration (Fig. 19).

The influence of Brownian motion parameter Nb on the temperature and concentration profiles is studied in Figs. 20 and 21. From these figures, we notice that an enhancement in the values of Nb gives rise to the temperature, while it causes a decrease in the nanoparticle concentration profile. Brownian motion is the random motion of nanoparticles suspended in the fluid, caused by the collision of nanoparticles with the fluid particles. An increment in the thermophoretic effect causes an increment in the Brownian motion effect which results in the rise of the temperature due to the increment in the kinetic energy.

Figures 22 and 23 illustrate the effect of thermophoresis parameter Nt on the temperature and the nanoparticles concentration profile. One can observe that temperature and concentration fields increase with an enhancement in Nt. Thermophoresis parameter plays an important role in the heat transfer flow. Thermophoresis force enhances when Nt is increased which tends to move the nanoparticles from the hot region to the cold and as a result the temperature and the boundary layer thickness increase.

Figures 24 and 25 shows the impact of the Lewis number (Le) on temperature and nanoparticle concentration profiles respectively. It is observed that the temperature increases by increasing Le while concentration decreases with an increase in the Lewis number.

Figure 26 envisages the activation energy (*E*) impact on concentration field. Graph elucidate that concentration profile increases for large value of *E*. The Arrhenius function deteriorations by snow balling the value of the activation energy, which outcomes in the promotion of the generative chemical reaction causing an improvement in the concentration field. Within the occurrence of low temperature and higher activation energy leads to a smaller reaction rate constant which slow down the chemical reaction. In this manner concentration profile boost up. Figure 27 shows that when chemical reaction rate increases, concentration profile strongly reduces because of high chemical reaction rate which fallouts solute boundary layer becomes thicker. When chemical reaction parameter increases steadily, the factor (1 + θ) *e*^−*E*/(1+θ)^ is enriches because of increase in values chemical reaction parameter.

The impact of the various physical parameters on the local Sherwood number, skin friction coefficient and local Nusselt number, mathematical results are achieved for Nb = 0.3, β = 0.5, Nt = 0.7, Pr = 0.71, Le = 0.6, M = 0.5, m = 0.2, Grx = 0.5, Grc = 0.5, Q = 0.5, and R = 1are enumerated as shown in Table 1. it is viewed that the skin-friction coefficient in x − direction decreases with an increase in the thermal Grashof number Gr, the mass Grashoff number Gm, Hall current parameter m, and Brownian motion parameter Nb, while it increases for the increasing value of magnetic parameter M, Heat source parameter, Radiation and Prandtl number Pr, and thermophoresis parameter Nt. A completely opposite behavior is recorded for the coefficient of the skin-friction in the z-direction. Nusselt number increases when the Hall current parameter m, thermal Grashof number, the mass Grashoff number, and Prandtl number, increase whereas it is reduced by increasing the value of Magnetic field parameter M, Heat source and radiation parameters. Sherwood number has increasing behavior for thermal Grashof number Gr, Magnetic field parameter M, Brownian motion parameter Nb, Heat source and radiation parameters and thermophoresis parameter Nt, while it has decreasing behavior for Grashoff number Gm and Prandtl number.

**Table 1:** Numerical values of ${\text{Re}}_{x}^{1/2} Cf_{x} ,\,\,\,\,\,\,\,{\text{Re}}_{x}^{1/2} Cf_{z} ,\,\,\,\,{\text{Re}}_{x}^{1/2} Nu_{x} ,\,\,\,\,{\text{Re}}_{x}^{1/2} Sh_{x}$.

Grx	Grc	Q	m	Nb	R	M	Pr	Nt	−2f″(0)	−2 g′(0)	−θ′(0)	−φ′(0)
0.5									1.2547	0.8521	0.5212	0.9514
1.0									0.9978	0.9125	0.5323	0.9912
1.5									0.7354	0.9542	0.5457	1.0245
	0.3								0.9875	0.8512	0.5032	0.1247
	0.6								0.8475	0.9852	0.5124	0.1108
	0.9								0.7125	1.2521	0.5785	0.9178
			1						1.5214	0.8521	0.3145	0.8852
			2						1.0214	0.9547	0.2978	0.7952
			3						0.8125	0.9985	0.2312	0.9452
				0.2					0.9512	0.9521	0.8452	0.5852
				0.4					0.8152	0.9612	0.8215	0.6124
				0.6					0.7125	0.9852	0.8032	0.6978
						0.5			0.9452	1.0254	0.9875	0.7852
						1.0			1.2454	0.9852	0.9125	0.8952
						1.5			1.4035	0.8512	0.8952	0.9452
							0.68		0.9785	1.0214	0.1254	0.9878
							0.71		0.9120	0.9852	0.1578	0.9452
							0.76		0.8452	0.9032	0.1987	0.9231
								0.3	1.5452	0.9852	0.8542	0.5120
								0.6	1.1254	0.9120	0.8125	0.5921
								0.9	0.8752	0.8962	0.7521	0.6120
		0.2							0.7124	0.9875	0.9542	0.9852
		0.4							0.7852	0.9452	0.9125	0.9745
		06							0.8125	0.9132	0.8657	0.9645
					0.5				0.8752	1.0875	0.7452	0.9124
					1.0				0.8952	0.9442	0.7365	0.9120
					1.5				0.9125	0.9114	0.7154	0.9102

For the authentication of the numerical method used, the results were compared with the previously obtained results Ibrahim and Anbessa^18^ for various values of parameters and it indicates an excellent accord as shown in Table 2.

**Table 2:** Comparison of − θ′ (0) for various values of Pr when Nb = 0.3, Nt = 0.7, P r = 0.71, Le = 0.6, M = 0.5, Gr = 0.5, Gm = 0.5, m = 0, Q = 0, R = 0, β = 0.

Pr	Ibrahim and anbessa^18^	Present values
0.01	0.019887	0.019125
0.72	0.808635	0.807785
1	1.000000	1.000000
3	1.923687	1.924785
10	3.720676	3.732452

## Conclusions

The influence of the Hall current and thermal radiation on the heat and mass transfer of nanofluid flowing across a linearly stretched sheet in the presence of Heat source/sink Thermophoresis and Brownian motion will be discussed in the present paper. The most significant accomplishments have been broken down into the following categories:

i. The resultant fluid velocity diminishes with increasing casson fluid parameter (β).
ii. The temperature increases as the Heat source/sink (Q) and Brownian motion parameter (Nb) values increase, but the concentration profile of nanoparticles decreases. The opposite behavior was observed for the case of Radiation parameter (R).
iii. The temperature and concentration fields intensify with a rise in the Thermophoresis parameter (Nt).
iv. The temperature and concentration profiles tend to fall when the Prandtl number (Pr) is raised.
v. The temperature increases by increasing Le while concentration decreases with an increase in the Lewis number
vi. The velocity increases with enhance of hall parameter (m), where as the reversal behavior has observed in the case of temperature and Concentration.

## Data Availability

All the data are clearly presented in the manuscript.

## List of symbols

a: A real constant
B_0_: Magnetic field strength
C: Concentration of fluid
Cf_x_: Skin friction coefficient along x-axis
Cf_z_: Skin friction coefficient along z-axis
C_w_: Concentration at wall
C_∞_: Ambient concentration
Cp: Specific heat
D_B_: Brownian diffusion coefficient
D_T_: Thermophoretic diffusion coefficient
g_c_: Gravitational acceleration
Grc: Mass Grashof number
Grx: Thermal Grashof number
k: Thermal conductivity
Le: Lewis number
M: Magnetic parameter
Nb: Brownian motion parameter
Nt: Thermophoresis parameter
Nux: Local Nusselt number
qr: Radiative heat flux
qw: Surface heat flux
Pr: Prandtl number
Rex: Local Reynolds number
R: Thermal radiation parameter
Shx: Local Sherwood number
T: Temperature of fluid
Tw: Temperature at wall
kr: Non dimensional chemical reaction parameter
K_E_: Dimensionless chemical reaction parameter
E: Activation energy parameter

## Subscripts and superscripts

w: Conditions on the wall
∞: Free stream conditions
